# Can we spend our way out of the AIDS epidemic? A world halting AIDS model

**DOI:** 10.1186/1471-2458-9-S1-S15

**Published:** 2009-11-18

**Authors:** Robert J Smith?, Jing Li, Richard Gordon, Jane M Heffernan

**Affiliations:** 1 Department of Mathematics and Faculty of Medicine, The University of Ottawa, 585 King Edward Ave, Ottawa, Ontario, Canada, K1N 6N5; 2 Department of Mathematics, The University of Ottawa, 585 King Edward Ave, Ottawa, Ontario, Canada, K1N 6N5; 3 Department of Radiology, University of Manitoba, Winnipeg, Manitoba, Canada, R3A 1R9; 4 Department of Mathematics & Statistics, York University, 4700 Keele St, Toronto, Ontario, Canada, M3J 1P3

## Abstract

**Background:**

There has been a sudden increase in the amount of money donors are willing to spend on the worldwide HIV/AIDS epidemic. Present plans are to hold most of the money in reserve and spend it slowly. However, rapid spending may be the best strategy for halting this disease.

**Methods:**

We develop a mathematical model that predicts eradication or persistence of HIV/AIDS on a world scale. Dividing the world into regions (continents, countries etc), we develop a linear differential equation model of infectives which has the same eradication properties as more complex models.

**Results:**

We show that, even if HIV/AIDS can be eradicated in each region independently, travel/immigration of infectives could still sustain the epidemic. We use a continent-level example to demonstrate that eradication is possible if preventive intervention methods (such as condoms or education) reduced the infection rate to two fifths of what it is currently. We show that, for HIV/AIDS to be eradicated within five years, the total cost would be ≈ $63 billion, which is within the existing $60 billion (plus interest) amount raised by the donor community. However, if this action is spread over a twenty year period, as currently planned, then eradication is no longer possible, due to population growth, and the costs would exceed $90 billion.

**Conclusion:**

Eradication of AIDS is feasible, using the tools that we have currently to hand, but action needs to occur immediately. If not, then HIV/AIDS will race beyond our ability to afford it.

## Introduction

There has been a sudden increase in the amount of money donors are willing to spend on the worldwide HIV/AIDS epidemic. Present plans are to hold most of the money in reserve and spend it slowly. However, rapid spending may be the best strategy for halting this disease. The purpose of this paper is to estimate how and when to best spend the available money to achieve halting HIV/AIDS as soon as possible.

In the year 2000, leaders from around the globe derived a set of goals aimed at making the world safer, healthier and more equitable. One of these goals was to halt and begin to reverse the global HIV/AIDS epidemic by 2015. To realise this goal, in 2006, world leaders pledged to move towards universal access to HIV prevention, treatment, care and support by 2010. In the past decade, there has been a marked decrease in the number of AIDS-related deaths worldwide and the percentage of individuals living with HIV has begun to plateau. Indeed, a number of highly infected countries have seen declines in the number of new HIV infections [1]. This success is owed largely to a six-fold increase in financing for HIV activities in low- and middle-income countries enabling better access to HIV treatments and care. However, the picture is not uniformly positive and some countries continue to see an increase in infections. Also, in spite of the improved access to HIV treatment and care, the majority of those who need antiretroviral therapies are not currently receiving them [1]. As a result, the epidemic is outpacing the rate at which these drugs are being delivered. In fact, the number of new infections is significantly greater than the increase in the number of people on antiretrovirals. Eradication of HIV/AIDS is therefore not achievable using the current funding model. In the absence of additional funding, we contend that a significant improvement in results can be achieved by optimising implementation, i.e. how donated funds are spent on drug delivery, educational programs and the distribution of HIV/AIDS prevention measures.

While estimates on spending have been made for many scenarios [2-4], these are usually projected in decades or generations. World events could overtake the HIV/AIDS epidemic on this time scale, or even be precipitated by it if it continues to grow [5-9]. Furthermore, donor fatigue [10] could return, or other crises (tsunamis, wars, etc) could divert presently committed funds. Is there a quick fix that could avoid passing the problem on to the next generation?

Consider, for example, Bill and Melinda Gates, who have made stopping AIDS the top priority of their foundation [11], with $60 billion available [12]. As an extreme case, for instance, ignoring the ability of economies to absorb so much cash [13], the Gates Foundation might spend all their money in two years, meeting the worldwide needs of $27 billion per year for all health needs of all developing countries [14] (including HIV/AIDS), with the hope that infrastructure would then be in place and the rest of the donors would pick up from there. The projected spending rate of the Gates Foundation is $3 billion per year [15], which is roughly comparable to the expected annual interest. It is being spread out over 20 years, more than doubling the duration of the epidemic, which has already lasted over 25 years. Something in between a jump start and a trickle would seem much more optimal than either of these extremes.

Furthermore, if we fail to spend at the optimal rate, the epidemic may blow up. Currently, the percentage of people living with HIV has stabilised; however, the overall number of people living with HIV has steadily increased [1]. The result is that the epidemic could become totally out of control or cost immensely more to bring under control. Many modeling studies have shown a bigger bang for the buck if the money is spent earlier in an epidemic [16,17], but none have investigated the optimal course of spending to bring an epidemic under control as rapidly as possible and avoid a growing gap [18].

A number of views have been expressed on how to tackle HIV/AIDS. One approach involves getting back to the fundamentals of epidemiology [19] and avoiding AIDS exceptionalism [20], i.e. treating HIV/AIDS just like other epidemics. Most people advocate a mixture of prevention and treatment options [17,21]. One worldwide single (but not magic) bullet approach that has been proposed would use the fact that highly active antiretroviral therapy (HAART) reduces infectivity:

"This optimistic population-based model shows that, in 45 years, HIV prevalence could be reduced by more than 70 times from more than 7 cases per 1000 people to less than 0.1 case per 1000. The number of HIV- infected people could be reduced from 38 million to less than 1 million. The cost of therapy would be about US $7 billion per year, with costs declining from $15 to $1 billion. Such a programme would be expected to cost $338 billion over 45 years" [22].

The average cost over the 45 years would be $7.5 billion per year.

Condoms offer another single-mode approach. Above an estimable and possibly attainable threshold, condoms could halt an HIV/AIDS epidemic [23]. If distributed to 1.5 billion men (half the male population of the world), at 100 condoms per person per year [24], and a cost of $0.02 each [25], total cost would be $3 billion per year. (Female condoms have their place, but are about ten times more expensive each.) For comparison, the USA spends $12 billion per year domestically on HIV/AIDS, plus $3 billion per year on HIV/AIDS research [26].

Condoms have the advantage of reducing other STIs (sexually transmitted infections), some of which enhance HIV infectivity. The threshold for decline of the epidemic is the product of compliance in use and condom effectiveness, and there is room for technical improvement of the latter [24]. The problems to be tackled are not simply behavioural, but also involve design, manufacturing, marketing and subsidisation [27]. Even in the behavioural realm, the emphasis on risk factors instead of collective behaviour may be counterproductive [28,29].

The HIV/AIDS epidemic is often spoken of in terms of reducing the spread [16], or achieving sustainable financing [30]. Decades-long investments have been made in vaccines [31] and more recently in microbicides [32]. These approaches are slow in coming, and do not take into account the greatly increased amount of cash now available, at least for the short term. The donor community has created the potential of stepping ahead of the exploding HIV/AIDS epidemic by pooling money faster than the disease is spreading. Our aim is to use mathematical modelling to find ways to spend the available money as fast, efficiently and effectively as possible to bring this epidemic to a full halt, now, while this transient advantage is still available.

A large number of mathematical models have been used to describe the epidemiology of HIV [33-46]. These range from the simple to the complicated, and have used a variety of techniques to analyse and determine conclusions. One such technique is that of metapopulation models: dividing space into distinct regions, within which disease transmission occurs (often at different rates) and with "travel" between regions allowing different regions to be connected. Such models have been used to describe HIV in a spatial context without resorting to partial differential equations [47-53].

This paper presents a simple, linear, metapopulation model that describes the world regions at a number of different scales (continent, country, etc). We use this model to show that interventions can lead to eradication, but such interventions must be applied globally, not locally, and that time is of the essence if our interventions are to succeed.

## An eradication model

In its simplest form, HIV infection can be described by the classical SI (susceptible-infected) model. The dynamics are not linear, depending, at a minimum, on the behaviour of susceptible (uninfected) individuals, as well as their interactions with those who are infected. While the SI model does not capture all the dynamics of HIV, it has been used to model the disease throughout its entire history [54-60].

Consider the two-dimensional SI model

Here, Λ is the rate of appearance of new susceptibles, *β *is the infection rate, *μ *is the background death rate and *γ *is the death rate due to disease.

This model has two equilibria,

The Jacobian matrix for this system is

with eigenvalues *λ *= -*μ*,  - *μ *- *γ*. It follows that the threshold for eradication [61] in the SI model is

The SI model, while it captures the dynamics of HIV spread in a population, is difficult to manipulate in a metapopulation scenario. We propose instead a linear approximation of this model. The approximation of the epidemic as linear in the number of infectives is clearly a coarse one. However, although this model structure does not capture the transient dynamics of infection and interaction, it nevertheless serves as a predictor for eradication.

This is because the major stability analysis carried out on such models - finding the basic reproductive ratio - consists of linearisation of the nonlinear models. Thus, a linear version of the model has the advantage of having the same invasion threshold, although it has the disadvantage of only describing local stability of the disease-free equilibrium.

Since , it follows that the one-dimensional model

will always overestimate the epidemic. We shall refer to this as the I-only model.

This model has only the trivial equilibrium *I *= 0. If the trivial equilibrium is stable, then all trajectories will approach it. If the trivial equilibrium is unstable, then solutions will increase without bound.

Although the total population without infection is not constant, it should nevertheless be noted that the I-only model has the eradication threshold

which is the same eradication threshold as the SI model (as expected). It follows that there will be eradication in the I-only model if and only if there is eradication in the SI model.

**Figure 1:**
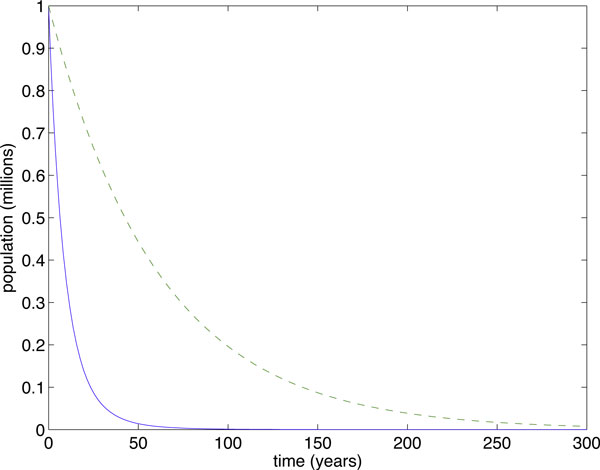
**When *R*_0 _< 1, both models lead to eradication**. When *R*_0 _< 1, both models lead to eradication. The SI model (solid curve) is always below the I-only model (dashed curve). In this case, *R*_0 _= 0.8575.

**Figure 2:**
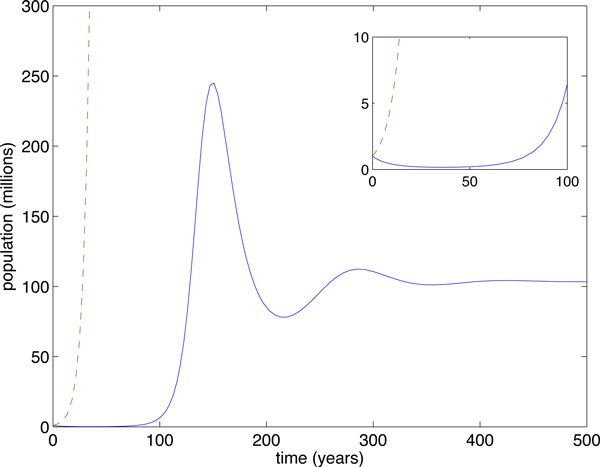
**When *R*_0 _> 1, both models lead to endemic disease**. When *R*_0 _> 1, both models lead to endemic disease. The I-only model (dashed curve) always overestimates the SI model (solid curve). In this case, *R*_0 _= 2.45.

To illustrate, we simulated two cases: *R*_0,*SI *_< 1 (Figure 1) and *R*_0,__*SI*_> 1 (Figure 2). Parameters used in the simulations were Λ = 20 people·years^-1^, *μ *=  years^-1^, *γ *=  years^-1^, with *β *= 0.00007 people^-1^years^-1 ^(Figure 1) and *β *= 0.0002 people^-1^years^-1 ^(Figure 2). Despite the fact that the transient dynamics are vastly different in the two models, the linear approximation has the same eradication threshold as the more accurate SI model and, furthermore, always overestimates the epidemic. It follows that, for eradication purposes, the simple, linear model should determine whether our control methods will be sufficient.

We now extend the *SI *model to a metapopulation model with *p *regions. For 1 ≤ *i *≤ *p*, we can write

*S*_*i *_= susceptible individuals in the *i*th region;

*I*_*i *_= infected individuals in the *i*th region;

Λ_*i *_= the rate of appearance of new susceptible individuals in the *i*th region;

*μ*_*i *_= background death rate in the *i*th region;

*γ*_*i *_= death rate due to disease in the *i*th region;

*n*_*ij *_= migration rate of susceptible individuals from *j*th region to *i*th region;

*m*_*ij *_= migration rate of infected individuals from *j*th region to *i*th region.

With these assumptions, we have *n*_*ii *_≡ *m*_*ii *_≡ 0. Then, for *i *= 1,..., *p*,

If the system (2) is well-posed, we can find a bound for the population of susceptibles in each patch. We can then extend the same idea to obtain the linear system of *I*-equations only. (See Appendix for details.)

From Theorem 1 in the Appendix, we have

Thus, the total population of susceptible individuals in the *i*th region is limited. So we can write

*π*_*i *_= *β *× (total population in the *i*th region without infection)

*d*_*i *_= *μ*_*i *_+ *γ*_*i*_,

where *d*_*i *_represents the total death rate in the *i*th region. Thus, for *i *= 1,..., *p*, the equations of the *I*-only model are

This can be rewritten as

where **I**(*t*) = (*I*_1_(*t*), *I*_2_(*t*),..., *I*_*p*_(*t*))^*T *^and

## Analysis

### A two-region example

The simplest nontrivial version of model (2) is the case when *p *= 2, which can be presented as follows:

The disease-free equilibrium for model (4) is  with

By using the next-generation matrix method [62], we can calculate the basic reproductive ratio for model (4),

where

This is the eradication threshold condition for this simple case *p *= 2. The case for *p *= 2 can be further simplified under different assumption of the travel (or migration) between the two regions. Here we list three possibilities.

#### Case 1

The two regions are isolated from each other. Then, for each of the two regions, we have, for *i *= 1, 2,

which has the eradication condition

#### Case 2

We only allow the susceptible individuals to travel (or migrate) between the two regions. Then we have

The disease-free equilibrium is again . However, the basic reproductive ratio (or the eradication condition for the disease) is given by

#### Case 3

The travel (or migration) between the two regions is unidirectional. Without loss of generality, we can assume that individuals only travel from Region 1 to Region 2. Thus, we have

The disease-free equilibrium for model (10) is , with

Using the next-generation matrix method again, we obtain the basic reproductive ratio

which is also the eradication condition for the disease in this case.

Comparing all of the above cases when allowing the travel (or migration) between the two regions, we know that all of the eradication conditions in (5), (9) and (11) are not as simple as (7), the case when the two regions are disconnected. All of them depend on the travel rate between the two regions to some extent. For example, for Case 2, denoting

calculation shows that

From this, one can derive conditions for the travel rate of susceptible individuals which can either help a disease which is otherwise dying out to persist locally or cause an otherwise partially persistent disease to persist globally in the two regions (see Appendix for the details). It follows that the disease must be reduced within each region and the effects of travel between regions must be accounted for. This explains why HIV eradication must be considered as a world problem, not just a problem for individual countries, or continents, to tackle independently.

#### Finding the eradication threshold condition *T*_0_

Since system (3) is linear, the stability of the disease-free equilibrium is determined by the sign of the real parts of the eigenvalues of the matrix **K**. The only equilibrium for the *I*-only model (3) is the trivial, disease-free equilibrium (0,...,0). The linear stability of the trivial equilibrium for (3) is determined by *s*(**K**), the stability modulus of **K**, which is defined as the maximal real part of all eigenvalues of the matrix **K**. Thus, we can define the overall eradication threshold as

The trivial equilibrium is globally asymptotically stable if *T*_0 _< 1 (for this linear system (3), the local stability is equivalent to global stability) and unstable if *T*_0 _> 1.

Noting that the right-hand side of *I*-equations in (2) is never bigger than the right-hand side of (3) and then by the standard comparison theorem (see [63,64]), one can show that *T*_0 _is also the condition to guarantee the stability of the disease-free equilibrium of system (2). This implies that HIV can be eradicated under the condition that *T*_0 _< 1, but it can not be eradicated under the condition that *T*_0 _> 1.

## A continent-level example

**Figure 3:**
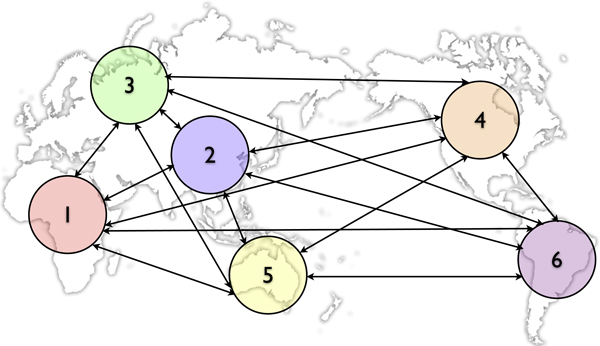
**A continent-level example**.

As a more realistic example, we divide the world into six continents: Africa, Asia, Europe, North America, Oceania and South America. Infection occurs within each continent and travel/immigration of infectives occurs between continents. See Figure 3.

In this case, model (2) is explicitly

Parameters for our model consist of death, birth, immigration and emigration rates at the continental level, as well as estimates for the number of sexual partners and infection rates. Immediate data describing these rates does not exist; however, we can determine many of these parameters at the continental level using country-specific data. The birth and death rates have been derived from crude birth and death rates per 1000 people. The continental birth and death rates are determined using weighted averages by country population (see Table 1). Data was taken from the CIA world factbook 2008 [65].

**Table 1:** Birth rates and death rates by continent [65].

	Population	Births/Popn	Birth Rate	Deaths/Popn	Death Rate
AF	954879489	33203380	0.0348	12625314	0.0132
AS	4043347897	76536061	0.0189	27947177	0.0069
EU	729546003	7321919	0.0100	8437988	0.0116
NA	527814776	8712554	0.0165	3798366	0.0072
OC	33970173	545421	0.0161	244250	0.0072
SA	383907961	6773241	0.0177	2354551	0.0061

Net immigration data is also reported in the CIA world factbook; however, this does not enable us to determine separate immigration and emigration rates. We can, however, determine continent-specific immigration and emigration rates from the number of foreign-born individuals by country. These are individuals who now reside in a particular country who stated that they were born in a foreign country on their census form. The Global Migrant Origin Database [66] gives the number of foreign-born individuals by country of origin and country of destination in the 2000 round of country censuses. This data has been summarised to determine the number of foreign born by continent; that is, individuals who now reside in a continent who were not born in that continent (see Table 2). Dividing Table 2 by the total number of foreign born gives the fraction of foreign-born individuals who migrated from continent A to continent B. If the number of immigrants each year is known, these fractions can be used to approximate the number of immigrants and emigrants by continent.

**Table 2:** Foreign born. Number of foreign born by continent of origin (vertical) and continent of destination (horizontal) [66].

Des/Ori	AF	AS	EU	NA	OC	SA
AF		15973	16987	4263	0	0
AS	286806		298431	13062	1621	32500
EU	5312095	3566013		1167954	252860	1337972
NA	1176374	9690228	8573379		353095	4466748
OC	221003	1380652	2470078	136804		76873
SA	16595	218415	1087422	197314	3324	

We use the information in Tables 1 and 2 to determine the eradication threshold *T*_0_. The off-diagonal entries of our matrix are the off-diagonal entries from Table 2. The diagonal entries of our matrix are calculated according to the formula

where Λ_*i *_is the influx of new susceptibles, *μ*_*i *_is the death rate (Table 1), *β *is the infection rate, *γ *is the AIDS-specific death rate, *η*_*ij *_is the number of individuals born in continent *j *residing on continent *i *per year (Table 2) and *P*_*i *_is the population of continent *i *(Table 1).

As an example, we choose *β *= 0.0002 people^-1^years^-1 ^and *γ *= 0.1 years^-1^, reflecting the fact that the typical time course of the disease is ten years. We calculate the influx of new susceptibles according to the formula

where *p*_*i *_is the continent's birth rate and *c *is an infected individual's annual number of (uninfected) sexual partners. We set *c *= 20; however, the factor  modifies an individual's annual number of sexual partners according to the growth (or decline) of the continent they are in. Thus, for Europe, the annual number of sexual partners is 17, whereas for Oceania the number is 44. These numbers are likely overestimates, but our intention is to be conservative in our approach.

If we now intervene and reduce *β *to 0.0002 x 2/5, then the overall threshold is . It follows that reducing the infection rate to 0.00008 will result in eradication.

**Figure 4:**
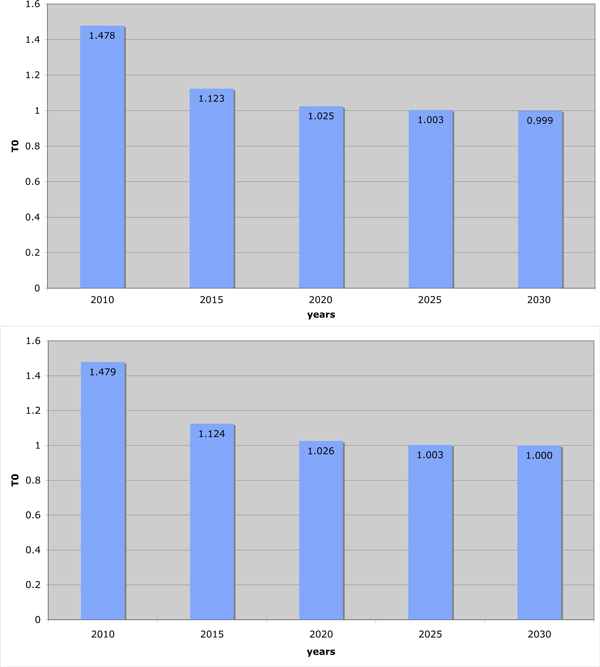
**Reduction in transmission**. The effect on the eradication threshold condition T_0 _of reducing the transmission rate over a twenty year period to two fifths of the current value by decreasing the infection rate from 2010 levels to two fifths its current value, in 2030. A. Assuming population demographics remain unchanged. B. Assuming 3% population growth per year. Population increases over time result in the inability to eradicate the disease using this technique.

Reducing *β *by three fifths is, of course, a mammoth task. Furthermore, this model implicitly assumes that such a reduction is instantaneous. To compare this to slowly reducing *β *over a period of decades, we assumed that *β *would be reduced to two fifths of its current value in twenty years' time, but that reductions would occur evenly throughout that time. However, we also included a population increase of 3% per year. The results are shown in Figure 4. In this case, the population "overruns" our goal. The overall *T*_0 _is not reduced below one, because the population in twenty years' time will be too high for a 3/5 reduction in the infection rate to lead to eradication.

## Costs

We now explore how much these intervention measures might cost. Interventions include changes in education, condoms, drugs and travel restrictions. The costs that can be applied to these different interventions vary greatly from region to region. For example, the cost of HAART in high-income regions is much greater than the cost of the same drug treatment regime in low-income regions. However, this lower cost is still expensive compared to the per-capita national health expenditures. In this section, we determine the costs that we are going to apply to our model so that we can determine an optimal way to spend donor money to eradicate HIV. We list some costs found in the literature associated with education, HIV testing, drug therapy and circumcision in Table 3. All costs are given in US dollars (USD).

**Table 3:** Costs of HIV/AIDS intervention methods.

Intervention	Cost (USD)	Reference
HIV testing in low-income countries	200-4000 per test	[74]
Combination antiretroviral therapy in high-income countries	10000-15000 per patient year	[75]
Combination antiretroviral therapy in low-income countries	350-4000 per patient year	[75-79]
Costs of monitoring viral load and cell count	25-100 per test	[76]
Health education	500-3000 per patient	[77,80,81]
Patient out-of-pocket expenses to receive medical care in a low-income area	60-250	[77]
Adult male cirumcision	40-100	[82]
Male condom	0.02 per condom	[25]

Along with the cost of delivering drugs to a patient, it is also important that we determine what benchmark of viral load and cell count we will use for initiation of therapy. It is obvious that the costs of delivering therapy and monitoring will increase if these are started at an earlier time in HIV infection. For example, it is common practice to initiate therapy in the developed world when CD4 counts are less than 350 cells/mm^3^. However, in the developing world, it is advised that treatment start when CD4 counts are less than 200 cell/mm^3^. In a study by Vijayaraghavan *et al. *[67], it was found that treating patients with HIV according to developed- versus developing-world guidelines is highly cost-effective and may result in substantial long-term savings. We choose to initiate individuals earlier in life for our cost analysis. This means that we will include a cost of therapy in the upper range of that listed in Table 3. However, we will only choose this cost from those for developing countries since the donor funds are applied mainly to the epidemic in the developing world.

Note that we assume that the costs of delivering therapy and health care to HIV infected individuals will stay relatively constant over a short period of time. Since the donor money will be spent over a span of a few years rather than decades, this assumption can be expected to give realistic results. If we were to determine the cost of such interventons over a long period of time, we must also include variables describing a decrease in the costs of therapy and testing as drug manufacturing becomes more efficient, the discovery and development of new drugs which will affect the cost of therapy, the increase in education and other medical expenses (since administrative costs and the payment of healthcare workers will increase over time), etc.

To effectively determine the cost of treatment, we also must include the possibility of the evolution of drug resistance [37]. It is difficult to put a dollar figure on the evolution of drug resistance, since it may occur during different periods of infection in different patients. In our cost analysis, we choose costs of treatment and testing in the upper range of that reported (see Table 3), since it is assumed that the evolution of resistance will increase the cost of treatment and require more tests to monitor a patient's viral load and cell count.

Referring back to model (3), it is evident that condoms and education will reduce *π*_*i*_, while treatment will reduce *π*_*i*_, but will also decrease *d*_*i*_. Education will include condom awareness, but may include other factors, such as abstinence and monogamy [68]. Travel/immigration restrictions will reduce *m*_*ij *_and *m*_*ji*_, but we do not expect that these will be realistic; past attempts to restrict movement based on travel or immigration have simply driven the epidemic underground [69].

Note that we assume a population growth of 3% per year. This corresponds to the maximum population growth rate reported by the CIA World Factbook. We use the maximum growth rate so that cost estimates give an upper bound of what may be realistic for donor spending. We also assume that HIV-positive cases will continue to increase at a rate of 3% per year, if no interventions are undertaken.

Our cost formula is thus

where *C *is the cumulative cost, *n *is the proportion of men who must ultimately receive condoms, *m *is the fraction of infected individuals who receive treatment, *r *is the timescale, ϵ is the (fixed) cost of distribution and education, and *θ *is the cost of treatment (including the cost of paid health workers, testing etc). Thus, in the first term of (17), there are 3 billion men, whose numbers increase by 3% every year, given 100 condoms each, at a cost of $0.02, with a fixed condom education campaign of $60 million. In the second term of (17), we assume that one tenth of infected individuals require treatment; thus, currently there are 3.3 million individuals requiring treatment, whose numbers increase by 3% every year, at an average cost of $2500 per patient per year.

If we provided condoms to 3/5 of all men over a period of twenty years and treated nobody, then the cumulative cost is

**Figure 5:**
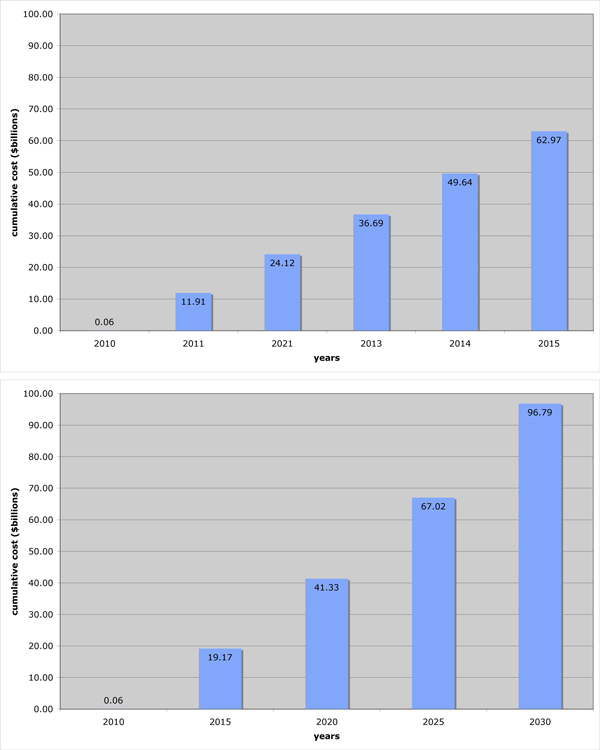
**Cumulative cost**. A. The cumulative cost of reducing the infection rate to two fifths of its current rate over the next five years and treating everybody who requires it. B. The cumulative cost of reducing the infection rate to two fifths of its current rate over the next twenty years (and treating nobody).

Thus, by steadily reducing the infection rate each year, the costs would blow up, way beyond the available funds and we would not even reach the eradication threshold. See Figure 5A.

If we treated everyone who requires treatment for twenty years, then the cumulative cost is

assuming no fixed costs of condom education.

If instead we aimed to treat 50% of people requiring treatment and provided condoms to 1/5 of all men within twenty years, then the cumulative cost is

Thus, treatment is significantly more expensive than prevention (as expected).

Conversely, suppose we aimed to reduce infection to two fifths of its current value and treat everybody infected over the next five years. Then the cumulative cost is

Thus, we could reach our eradication threshold within five years, provide treatment to everyone who needs it and stay within our $60 billion budget (plus interest). See Figure 5B.

## Discussion

We have provided a method for predicting treatment and prevention levels necessary to eradicate HIV/AIDS, based on population and immigration data at the continental level. This method is easily applicable to a finer scale, such as country-level data. The mathematical model is linear, which allows it to be easily generalised. We have also developed a formula to estimate the cost of some of these interventions.

It should be stressed that our model does not attempt to quantify the prevalence of the disease or the time course of infection. The model is a predictor of eradication only and thus should not be used in other contexts. However, it provides us with insight into the degree of intervention measures that are needed to eradicate the disease: an across-the-board reduction in the infection rate by a factor of three fifths or more, and antiretroviral treatment for everyone who requires it.

These are steep requirements that will cost a lot of money. However, we have demonstrated that spending only some of the money now and saving the rest for the future will result in a loss of ability to eradicate the disease, using existing estimates. We have clear, specific goals and they need to be acted upon immediately.

Our model also demonstrates the folly of focusing locally. Even if the basic reproductive ratio in all continents were reduced below one, the disease could still be sustained by travel or immigration (see Appendix). We note that travellers/immigrants do not present a high risk factor at present, but they may be a significant obstacle when the disease is close to eradication. This is why we must consider HIV/AIDS from a global perspective.

It should be noted that, in addition to our model's obvious shortcomings, our parameter estimates also have limitations. In particular, the data we use currently assumes that the disease-specific death rate is negligible, compared to the background death rate. While this may be approximately true in some western countries, it is clearly not the case in Africa [70]. However, while the numbers used here are illustrative, our calculations of *T*_0 _are based on analytical thresholds. Thus, the sensitivity of the results to our parameter choices is easily measured: variations in the data in Tables 1 and 2, as well as formulas (15) and (16) allows similar calculations to be performed at other scales than continental (e.g. country-level).

Another assumption that should be noted is that we assume that travellers/immigrants receive no intervention help, such as education or treatment. This may be likely if foreign nationals receive no health care and have limited access to education, but will not be universally true. However, in each case we have overestimated the severity of the disease, in order to provide a strict criterion: if our overall *T*_0 _- calculated from the linear model with the overestimated parameters - is less than one, then we will have eradication. Our immigration/emigration data did not include tourism, especially sex tourism [71] which potentially adds a huge effect to the impact of travellers.

Although the cost of a condom is relatively cheap compared to other intervention measures, it is necessary to acknowledge the fact that a condom taken does not mean a condom used. Condom promotion requires a well- developed program infrastructure with excellent logistic competence of its staff which may be difficult to achieve. Many areas, especially rural and remote villages, are not accessible; for example, the healthcare system covers less than 50% of the population in many sub-Saharan African countries. In addition, the effectiveness of condoms in HIV prevention is much less than 100%, perhaps mostly due to condom failure [24,72]. In addition, there are civil-war zones, such as eastern Congo, where services cannot be provided.

This further limits the lack of accessibility to reach out universally to all parts of the populations. However, model (3) has the property that decreasing *T*_0 _will still be beneficial. Thus, even if intervention methods are imperfect, a large-scale attempt at eradication will still be enormously effective.

Similarly, although we have estimated treatment costs, we have used aggregate numbers that do not explicitly take into account many of the specific costs associated with treatment, such as healthcare worker training. It has been shown that 100% HAART coverage in sub-Saharan Africa could be achieved in 10 years, if the number of healthcare workers were doubled every year [73], but this becomes expensive quite quickly.

There are, of course, many other intervention methods that could be examined, such as circumcision, STD control etc. These methods could be applied together with the proposed interventions; e.g. condom promotion could be combined with syphilis treatment without adding lots of additional costs. As we have learned from family planning programs, the more methods are offered to a client and the greater the choice is, the more likely the client is using one. This is likely to apply for HIV/AIDS control as well; more methods are advantageous and more likely to lead to positive results. Recently, UNAIDS has also made this proposition to promote as many methods as are available and proven effective.

It is currently advised that countries with different epidemic patterns will require different national strategies for implementing effective HIV prevention programs. Countries classified as low-level, concentrated, generalised or hyperendemic should concentrate their efforts on different national strategies.

In our model, we have applied the same strategy to all regions and each region includes countries with one or more of these classifications. We note, however, that the education component could be specific to each continent, or to each country.

Furthermore, although model (3) predicts eradication if *T*_0 _< 1, the threshold also has the property that lower values are beneficial. Thus, even if actual eradication cannot be achieved (as may be the case, in reality), intervention methods such as those mentioned here that reduce *T*_0 _will nevertheless be beneficial.

Thus, for example, if some countries make significant inroads against the disease, it will still have a global benefit even if eradication does not occur.

We view this model as a starting point.  It provides a criterion for eradication, but we believe that a more accurate model, one that reflected the time course of the disease in all regions, is achievable. However, such a model requires accurate input from all countries; ideally, it could be updated as more data becomes available, or as new intervention methods are developed. Disease eradication has occurred on a global scale before, as happened with smallpox and is currently underway for polio. While the mechanics of organising the international community in this way are beyond the scope of this paper, given the scale of the problem and the immediacy of the required solution, we believe that this model can provide us with an easy-to-grasp way of understanding what needs to be done and when to do it. For the former, our strategy involves harnessing all existing intervention techniques in significant strength. For the latter, the time is now.

## Appendix

### Well-posedness of system (2)

Consider the system (2) with the following initial conditions:

Define

and

The following theorem assures that the system is well-posed.

#### Theorem 1

*If *(*S*_10_,⋯, *S*_*p*0_, *I*_10_,⋯, *I*_*p*0_) ∈ *D*_*L*_, *then*, *for any L *>L*, *the set D*_*L *_*is positively invariant for solutions of (2) with (18).*

#### Proof

We first show that the solutions of (2) with (18) are nonnegative. For this purpose, let us rewrite the system (2) as follows:

where **S**(*t*) = (*S*_1_(*t*),⋯, *S*_*p*_(*t*))^*T*^, **I**(*t*) = (*I*_1_(*t*),⋯, *I*_*p*_(*t*))^*T*^, **Λ **= (Λ_1_,⋯,Λ_*p*_)^*T*^,

and

with , for *i *= 1,..., *p*. Noting that the off-diagonal matrix elements of **A**(*t*) are nonnegative, we conclude that the entries of the matrix  are all nonnegative. Indeed, let  and rewrite **A**(*t*) as

where **E **denotes the *p *× *p *identity matrix. Then all entries of (*t*) are nonnegative, and hence so are entries of . We also have

Noting that the scalar matrix -*G*(*t*)**E **is commutative with any *p × p *matrix (hence with (*t*)), we have

implying that all entries of  are nonnegative. Now, from (19), we have

Similarly, for any *t *≥ 0, all entries of  are nonnegative. Now, (20) leads to

implying **I**(*t*) ≥ 0 for *t *≥ 0.

Finally, we show that *S*_*i*_(*t*) and *I*_*i*_(*t*) are bounded for *t *≥ 0 and *i *= 1,..., *p*. Let *N*(*t*) = *S*_1_(*t*) + ⋯ + *S*_*p*_(*t*) + *I*_1_(*t*) + ⋯ + *I*_*p*_(*t*). By the nonnegativity of *S*_*i*_(*t*) and *I*_*i*_(*t*), for *i *= 1,..., *p*, we

have

This implies that *N*(*t*) is bounded with an upper bound ; hence, so are *S*_*i*_(*t*) and *I*_*i*_(*t*)

for *t *≥ 0 and *i *= 1,..., *p*. This completes the proof.   □

### Comparison of Case 1 and 2 in Section 3.1

It is obvious from the formulas in (13) that  and  reflect the influence of travel of susceptible individuals between the two patches, and hence may be called the *travel-modified basic reproductive ratios *for Region 1 and Region 2, respectively. The following observations are direct consequences of (12)-(13).

Their verifications are straightforward and are thus omitted.

**(A1) **Assume  < 1 and  < 1. If *n*_12 _> 0 and *n*_21 _> 0 satisfy either

or

then  > 1 and  < 1. By symmetry, the conditions parallel to the above can lead to  < 1 and  > 1. Here, and in the following, we omit such parallel conditions.

**(A2) **Assume  < 1 and  > 1. If *n*_12 _> 0 and *n*_21 _> 0 satisfy either

or

then  > 1 but  > 1.

The biological meanings of **(A1)-(A2) **can be obtained in terms of the threshold condition meaning of  and . For example, **(A1) **implies that travel of the susceptible individuals can help an otherwise dying out disease persist locally. Roughly speaking, a larger inflow of susceptible individuals favours the persistence of the disease in the region. **(A2) **shows that appropriate travel rates may cause an otherwise partially persistent disease to persist globally in both regions.

## Competing interests

The authors declare that they have no competing interests

## Authors' contributions

RJS wrote the manuscript and performed numerical simulations. JL wrote the appendix. RG wrote the introduction. JMH collected the data. All authors read and approved the final manuscript.
